# Semi-Parametric Estimation Using Bernstein Polynomial and a Finite Gaussian Mixture Model

**DOI:** 10.3390/e24030315

**Published:** 2022-02-23

**Authors:** Salima Helali, Afif Masmoudi, Yousri Slaoui

**Affiliations:** 1 Mathematics Laboratory, Angers University, 49100 Angers, France; salima.helali@univ-angers.fr; 2 Probability and Statistics Laboratory, Sfax University, Sfax 3029, Tunisia; afif.masmoudi@fss.usf.tn; 3 Mathematics and Applications Laboratory, Poitiers University, 86073 Poitiers, France

**Keywords:** asymptotic properties, Bernstein polynomial, EM algorithm, Gaussian mixture model, kernel estimator, shrinkage estimator

## Abstract

The central focus of this paper is upon the alleviation of the boundary problem when the probability density function has a bounded support. Mixtures of beta densities have led to different methods of density estimation for data assumed to have compact support. Among these methods, we mention Bernstein polynomials which leads to an improvement of edge properties for the density function estimator. In this paper, we set forward a shrinkage method using the Bernstein polynomial and a finite Gaussian mixture model to construct a semi-parametric density estimator, which improves the approximation at the edges. Some asymptotic properties of the proposed approach are investigated, such as its probability convergence and its asymptotic normality. In order to evaluate the performance of the proposed estimator, a simulation study and some real data sets were carried out.

## 1. Introduction

Density estimation is a widely adopted tool for multiple tasks in statistical inference, machine learning, visualization and exploratory data analysis. Existing density estimation algorithms can be categorized into either parametric, semi-parametric, or non-parametric approaches. In the non-parametric framework, several methods have been set forward for the smooth estimation of density and distribution functions. The most popular one, called kernel method, was introduced by [1]. The advances were carried out by [2] to estimate a density function. The reader is recommended to consult the paper [3] for an introduction to several kernel smoothing techniques. However, kernel methods display estimation problems at the edges, when we have a random variable *X* with density function *f* supported on a compact interval. Moreover, if $X_{1},\ldots ,X_{n}$ is a sample with the same density *f*, it is well known, in non-parametric kernel density estimation, that the bias of the standard kernel density estimator

(1) $$\begin{array}{ccc} {\hat{f}}_{n}\left(x\right) & = & \frac{1}{nh_{n}}\sum_{i=1}^{n}K\left(\frac{x-X_{i}}{h_{n}}\right), \end{array}$$

is of a larger order near the boundary than that in the interior, where *K* is a kernel (that is, a positive function satisfying ∫K(x)dx=1) and ($h_{n}$) is a bandwidth (that is, a sequence of positive real numbers that goes to zero). Let us now suppose that *f* has two continuous derivatives everywhere and that, as n→∞, $h=h_{n}\to 0$ and nh→0. Let x=ph for p>0. Near the boundary, the expression of the mean and the variance are indicated as

$$E\left[{\hat{f}}_{n}\left(x\right)\right]≃f\left(x\right)\int_{-\infty }^{p}K\left(x\right)dx-f^{\prime }\left(x\right)\int_{-\infty }^{p}xK\left(x\right)dx+\frac{1}{2}h^{2}f^{\prime \prime }\left(x\right)\int_{-\infty }^{p}x^{2}K\left(x\right)dx,$$

and

$$Var\left[{\hat{f}}_{n}\left(x\right)\right]≃{\left(nh\right)}^{-1}f\left(x\right)\int_{-\infty }^{p}K^{2}\left(x\right)dx.$$

These bias phenomena are called boundary bias. Numerous authors have elaborated methods for reducing these phenomena, such as data reflection [4], boundary kernels [5,6,7], local linear estimator [8,9], use of beta and gamma kernels [10,11] and bias reduction [12,13]. For a smooth estimator of a density function *f* with finite known support, there have been several methods, such as Vitale’s method [14], which is based on Bernstein polynomials and expressed as

(2) $$\begin{array}{ccc} {\tilde{f}}_{1,n,m}\left(x\right) & = & m\sum_{k=0}^{m-1}\left[F_{n}\left(\frac{k+1}{m}\right)-F_{n}\left(\frac{k}{m}\right)\right]b_{k}\left(m-1,x\right), \end{array}$$

where $F_{n}$ is the empirical distribution function and $b_{k}\left(m,x\right)=C_{m}^{k}x^{k}{\left(1-x\right)}^{m-k}$ is the Bernstein polynomial. This estimator was investigated in the literatures [15,16,17,18] and, more recently, by [12,19,20].

Within the parametric framework, it is noteworthy that the Gaussian mixture model can be used to estimate any density function, without any problem of estimation on the edge. This refers to the fact that the set of all normal mixture densities is dense in the set of all density functions under the $L^{1}$ metric [21]. The investigation of mixture models stands for a full field in modern statistics. It is a probabilistic model introduced by [22] to illustrate the presence of subpopulations within an overall population. It has been developed so far by various authors, such as [23]. It is used for data classification and it provides efficient approaches of model-based clustering. The authors of [24] demonstrated that, when a Gaussian mixture model is used to estimate a density non-parametrically, the density estimator that uses the Bayesian information criterion (BIC) of [25] to select the number of components in the mixture is consistent [26].

However, we obtain the non-parametric kernel estimate of a density if we fit a mixture of *n* components in equal proportions 1/n, where *n* is the size of the observed sample. As a matter of fact, it can be inferred that mixture models occupy an interesting niche between parametric and non-parametric approaches to statistical estimation.

More recently, in the parametric context, [27] proposed a parametric model using Bernstein polynomials with positive coefficients to estimate the unknown density function *f*; this estimator is defined as follows:

(3) $$\begin{array}{ccc} f_{B}\left(x,p_{m}\right) & = & \sum_{i=1}^{m}{\hat{p}}_{mi}B_{mi}\left(x\right), \end{array}$$

where $B_{mi}\left(x\right)=\left(m+1\right)b_{i}\left(m,x\right)$, for i=0,…,m, $p_{m}={\left(p_{m1},\ldots ,p_{mm}\right)}^{T}$ ($p_{mi}\geq 0$, i=1,…,m, $\sum_{i=1}^{m}p_{mi}\leq 1$) and ${\hat{p}}_{mi}$ are the estimators of the parameters $p_{mi}$, obtained by the Expectation Maximization (EM) algorithm as follows:

$$p_{mi}^{\left(s+1\right)}=\frac{1}{n}\sum_{j=1}^{n}\frac{p_{mi}^{\left(s\right)}B_{mi}\left(x_{j}\right)}{\sum_{k=0}^{m}p_{mk}^{\left(s\right)}B_{mk}\left(x_{j}\right)},\;i=0,\ldots ,m;\;s=0,1,\ldots$$

with ${\hat{p}}_{mi}=\lim_{s\to \infty }p_{mi}^{\left(s\right)},$ for i=1,…,m. The proposed method gives a consistent estimator in $L^{2}$ distance under some conditions.

The problem at the edge does not arise for the parametric model. For this reason, the basic idea of this work is to consider a shrinkage method using Bernstein (Vitale’s estimator) and Gaussian mixture estimators, to construct a shrinkage density estimator, in order to improve the approximation at the edge. A shrinkage estimator is a convex combination between estimators [28]. Basically, this implies that a naive or raw estimate is improved by combining it with other information.

The remainder of this paper is organized as follows: In the next section, we recall some intrinsic properties of the classical EM algorithm in the context of the Gaussian mixture parameter estimation. In Section 3, we introduce a new semi-parametric estimation approach based on the shrinkage method using Bernstein polynomials and Gaussian mixture densities. In Section 4, the consistency of the proposed estimator is exhibited, as well as its asymptotic normality.

Section 5 highlights a simulation study that compares the performance of the proposed approach with the Bernstein estimator, the standard Gaussian kernel estimator and Guan’s estimator. The closing Section 6 crowns the whole work, wraps the conclusion and provides new perspectives for future work.

## 2. Background

### The Gaussian Mixture Model and Em Algorithm

Let us consider $X=(X_{1},\ldots ,X_{n})$, a sequence of independent and identically distributed (i.i.d.) with common Gaussian mixture density defined by

(4) $$\begin{array}{ccc} g(x|\theta ) & = & \sum_{k=1}^{K}\pi_{k}N\left(\mu_{k},\sigma_{k}\right)\left(x\right), \end{array}$$

where

$$\theta =\left(\pi ,\mu ,\sigma \right)=\left(\pi_{1}\ldots ,\pi_{K},\mu_{1}\ldots ,\mu_{K},\sigma_{1},\ldots ,\sigma_{K}\right),$$

satisfies

$$0\leq \pi_{k}\leq 1,\sum_{k=1}^{K}\pi_{k}=1,\mu_{k}\in R,\sigma_{k}>0,\;\mathrm{for}\;k=1,\ldots ,K\;\mathrm{where}\;K>0,$$

and

$$N\left(\mu ,\sigma \right)\left(x\right)=\frac{1}{\sigma \sqrt{2\pi }}\exp\left(-\frac{{\left(x-\mu \right)}^{2}}{2\sigma^{2}}\right).$$

Finally, for each observed data point $X_{i}$, we associate a component label vector $Z_{i}$ in order to manage the data clustering. This random vector $Z_{i}={\left(Z_{ik}\right)}_{1\leq k\leq K}$ is defined such that $Z_{ik}=1$ if the considered observation $X_{i}$ is drawn form the $k^{kt}$ component of the mixture and $Z_{ik}=0$ otherwise. Consequently, $Z_{i}$ is distributed as a multivariate Bernoulli distribution with vector parameters $\left(\pi_{1},\ldots ,\pi_{K}\right)$ as follows:

$$P\left(Z_{i}=z_{i}\right)=\prod_{k=1}^{K}\pi_{k}^{z_{ik}}.$$

The EM algorithm is a popular tool in statistical estimation problems involving *incomplete data* or problems which can be posed in a similar form, such as the mixture parameters estimation [23,29]. In the EM framework, $\left(X_{1},\ldots ,X_{n},Z_{1},\ldots ,Z_{n}\right)$ corresponds to the complete data and $\left(Z_{1},\ldots ,Z_{n}\right)$ stand for the hidden data. Hence, the complete-data log-likelyhood is expressed by

(5) $$\begin{array}{ccc} L(X_{1},\ldots ,X_{n},Z_{1},\ldots ,Z_{n},\theta ) & = & \sum_{i=1}^{n}\sum_{j=1}^{K}Z_{ij}\left[\log\left(\pi_{j}\right)+\log\left(N\left(\mu_{j},\sigma_{j}\right)\left(X_{i}\right)\right)\right]. \end{array}$$

The two steps of the EM algorithm, after *l* iterations, are the following:

**(i)**  *E-step*: The conditional expectation of the complete-data log-likelyhood given the observed data, using the current fit $\theta^{\left(l\right)}$, is defined by
  (6) $$\begin{array}{ccc} \varphi \left(\theta |\theta^{\left(l\right)}\right) & = & E_{\theta^{\left(l\right)}}\left(L\left(X_{1},\ldots ,X_{n},Z_{1},\ldots ,Z_{n},\theta \right)|X_{1},\ldots ,X_{n}\right). \end{array}$$
  The posterior probability that $X_{i}$ belongs to the jth component of the mixture at the lth iteration, is expressed as
  (7) $$\begin{array}{c} \tau_{ij}^{\left(l\right)}=E_{\theta^{\left(l\right)}}\left(Z_{ij}|X_{1},\ldots ,X_{n}\right)=\frac{\pi_{j}^{\left(l\right)}N\left(\mu_{j}^{\left(l\right)},{\left(\sigma^{2}\right)}_{j}^{\left(l\right)}\right)\left(X_{i}\right)}{\sum_{h=1}^{K}\pi_{h}^{\left(l\right)}N{\left(\mu_{h}^{\left(l\right)},(\sigma_{h}^{2}\right)}^{\left(l\right)}\left(X_{i}\right)}. \end{array}$$
  Finally, we obtain
  (8) $$\begin{array}{ccc} \varphi \left(\theta |\theta^{\left(l\right)}\right) & = & \sum_{i=1}^{n}\sum_{j=1}^{K}\tau_{ij}^{\left(l\right)}\left[\log\left(\pi_{j}\right)+\log\left(N\left(\mu_{j},\sigma_{j}\right)\left(X_{i}\right)\right)\right]. \end{array}$$
**(ii)**  *M-step*: It consists of a global maximization of $\varphi \left(\theta |\theta^{\left(l\right)}\right)$ with respect to θ.
  (9) $$\begin{array}{ccc} \theta^{\left(l+1\right)} & = & \arg\max_{\theta }\varphi \left(\theta |\theta^{\left(l\right)}\right). \end{array}$$
  The updated estimates are stated by
  (10) $$\begin{array}{c} \pi_{j}^{\left(l+1\right)}=\frac{1}{n}\sum_{i=1}^{n}\tau_{ij}^{\left(l\right)}, \end{array}$$
  (11) $$\begin{array}{ccc} \mu_{j}^{\left(l+1\right)} & = & \frac{\sum_{i=1}^{n}\tau_{ij}^{\left(l\right)}X_{i}}{\sum_{i=1}^{n}\tau_{ij}^{\left(l\right)}}, \end{array}$$
  (12) $$\begin{array}{ccc} {\left(\sigma_{j}^{2}\right)}^{\left(l+1\right)} & = & \frac{\sum_{i=1}^{n}\tau_{ij}^{\left(l\right)}{\left(X_{i}-\mu_{j}^{\left(l+1\right)}\right)}^{2}}{\sum_{i=1}^{n}\tau_{ij}^{\left(l\right)}}. \end{array}$$

We repeat these two steps until $\left|\left|\theta^{\left(l+1\right)}-\theta^{\left(l\right)}\right|\right|<\epsilon ,$ where ϵ is a fixed threshold of convergence. The convergence properties of the EM algorithm have been investigated by [29] and by [30]. Relying upon Jensen’s inequality, it can be noticed that, as $\varphi \left(\theta |\theta^{\left(l\right)}\right)$ increases, the log-likelihood function also increases [29]. Consequently, the EM algorithm converges within a finite iteration number and gives the parameters’ maximum likelihood estimates. Therefore, under some conditions and according to [29], we have

(13) $$\begin{array}{c} \lim_{l\to \infty }\pi_{j}^{\left(l\right)}={\hat{\pi }}_{j},\;\lim_{l\to \infty }\mu_{j}^{\left(l\right)}={\hat{\mu }}_{j}\;\mathrm{and}\;\lim_{l\to \infty }{\left(\sigma_{j}^{2}\right)}^{\left(l\right)}={\hat{\sigma^{2}}}_{j}\;\mathrm{almost}\;\mathrm{surely}\;\left(a-s\right). \end{array}$$

In what follows, $\hat{\theta }=\left({\hat{\pi }}_{1},\ldots ,{\hat{\pi }}_{K},{\hat{\mu }}_{1},\ldots ,{\hat{\mu }}_{K},{\hat{\sigma }}_{1},\ldots ,{\hat{\sigma }}_{K}\right)$.

## 3. Proposed Approach

The proposed semi-parametric approach rests upon the *shrinkage* combination between the Gaussian mixture model and the Bernstein density estimators using the EM algorithm for the parameter estimations. The literature on shrinkage estimation is enormous. From this perspective, it is noteworthy to mention the most relevant contributions. The authors of [28] were the first to introduce the classic shrinkage estimator. The authors of [31] provided theory for the analysis of risk. Oman [32,33] developed estimators which shrink Gaussian density estimators towards linear subspaces. An in-depth investigation of shrinkage theory is displayed in Chapter 5 of [34].

The proposed semi-parametric approach based upon estimating the density function *f* relies on the same principle of Stein’s works and there are two aspects along this line. The first setting is non-parametric in the sense that we do not assume any parametric form of the density. The non-parametric setting is very important as it allows us to perform statistical inference without making any assumption on the parametric form of the true density *f*. The second setting is to consider the Gaussian mixture model as a parametric estimator of the unknown density *f*.

In what follows, we consider $X_{1},\ldots ,X_{n}$ a sequence of i.i.d. random variables having a common unknown density function *f* supported on [0,1]. We here develop a shrinkage method to estimate the density function, which is divided into the following three steps:

Step 1 We consider the Bernstein estimator of the density function *f*, which is defined as
  (14) $$\begin{array}{ccc} {\tilde{f}}_{1,n,m}\left(x\right) & = & m\sum_{i=0}^{m-1}\left[F_{n}\left(\frac{i+1}{m}\right)-F_{n}\left(\frac{i}{m}\right)\right]b_{i}\left(m-1,x\right) \end{array}$$
Step 2 In view of (13), we consider the Gaussian mixture density as an estimator of the density function *f*, given by
  (15) $$\begin{array}{c} {\tilde{f}}_{2,n}\left(x\right)=\sum_{k=1}^{K}{\hat{\pi }}_{k}N\left({\hat{\mu }}_{k},{\hat{\sigma }}_{k}\right)\left(x\right), \end{array}$$
  where ${\hat{\mu }}_{k}$, ${\hat{\sigma }}_{k}$ and ${\hat{\pi }}_{k}$ are estimated by the EM algorithm defined in (13).
Step 3 We consider the shrinkage density estimator ${\hat{f}}_{n,m}$ form defined by
  $${\hat{f}}_{n,m}\left(x\right)=\lambda {\tilde{f}}_{1,n,m}\left(x\right)+\left(1-\lambda \right){\tilde{f}}_{2,n}\left(x\right),$$
  and we use the EM algorithm to estimate the parameter λ∈[0,1] of the proposed model.

By the same way as considered in Section 2, the two steps of the EM algorithm, after *t* iterations, are denoted in terms of the following:

**1.**  *E-step*: The conditional expectation of the complete-data log-likelihood given the observed data, using the current $\lambda^{\left(t\right)}$, is provided by
  $$\begin{array}{ccc} Q(\lambda |\lambda^{\left(t\right)}) & = & \sum_{i=1}^{n}E_{\lambda^{\left(t\right)}}\left(W_{i1}|X_{i}\right)\log{\tilde{f}}_{1,n,m}\left(X_{i}\right)+E_{\lambda^{\left(t\right)}}\left(W_{i2}|X_{i}\right)\log{\tilde{f}}_{2,n}\left(X_{i}\right), \end{array}$$
  where $W_{i}=\left(W_{i1},W_{i2}\right)$ is a discrete random vector, following a multivariate Bernoulli distribution with vector parameters (λ,1−λ). Using Bayes’s formula, we obtain the posterior probability in the tth iteration denoted by
  $$\begin{array}{ccc} {\bar{\tau }}_{i1}^{\left(t\right)} & = & \frac{{\tilde{f}}_{1,n,m}\left(X_{i}\right)\lambda^{\left(t\right)}}{\lambda^{\left(t\right)}{\tilde{f}}_{1,n,m}\left(X_{i}\right)+\left(1-\lambda^{\left(t\right)}\right){\tilde{f}}_{2,n}\left(X_{i}\right)}, \end{array}$$
  and
  $$\begin{array}{ccc} {\bar{\tau }}_{i2}^{\left(t\right)} & = & \frac{{\tilde{f}}_{2,n}\left(X_{i}\right)\lambda^{\left(t\right)}}{\lambda^{\left(t\right)}{\tilde{f}}_{1,n,m}\left(X_{i}\right)+\left(1-\lambda^{\left(t\right)}\right){\tilde{f}}_{2,n}\left(X_{i}\right)}=1-{\bar{\tau }}_{i1}^{\left(t\right)}. \end{array}$$
**2.**  *M-step*: It consists of a global maximization of $Q(\lambda |\lambda^{\left(t\right)})$ with respect to λ.
  $$\lambda^{\left(t+1\right)}=\arg\max_{\lambda }Q\left(\lambda |\lambda^{\left(t\right)}\right).$$
  The updated estimate of λ is indicated by
  $$\lambda^{\left(t+1\right)}=\frac{1}{n}\sum_{i=1}^{n}{\bar{\tau }}_{i1}^{\left(t\right)}.$$

The estimation of λ is obtained from by iterating the EM algorithm until convergence.

(16) $$\begin{array}{c} \lim_{t\to \infty }\lambda^{\left(t\right)}=\hat{\lambda }. \end{array}$$

Therefore, the proposed estimator of the density function *f* is defined by

(17) $$\begin{array}{ccc} {\hat{f}}_{n,m}\left(x\right) & = & \hat{\lambda }{\tilde{f}}_{1,n,m}\left(x\right)+\left(1-\hat{\lambda }\right){\tilde{f}}_{2,n}\left(x\right). \end{array}$$

Basically, it is a shrinkage estimator that shrinks the Bernstein estimator towards the Gaussian mixture density by a specified amount of λ. If λ=1, the estimator ${\hat{f}}_{n,m}$ reduces to the Bernstein estimator ${\tilde{f}}_{1,n,n}$.

## 4. Convergence

In this section, we derive some asymptotic properties of the proposed estimator ${\hat{f}}_{n,m}$ when the sample size tends to infinity. First, we assume that λ and *K* are fixed. The following proposition gives the probability convergence of the proposed estimator ${\hat{f}}_{n,m}$.

**Proposition** **1** (Probability convergence)**.** *If $m=o\left(n/\log(n)\right)$, then, for x∈[0,1], we have*

$${\hat{f}}_{n,m}\left(x\right)\underset{n,m\to +\infty }{\overset{P}{⟶}}\lambda f\left(x\right)+\left(1-\lambda \right)f_{2}\left(x\right),$$

*where $f_{2}\left(x\right)=\sum_{j=1}^{K}\pi_{j}N\left(\mu_{j},\sigma_{j}^{2}\right)\left(x\right)$, $\pi_{j}=E\left(Z_{1j}\right),$ $\mu_{j}=E\left(X_{1}|Z_{1j}=1\right),$ $\sigma_{j}^{2}=Var\left(X_{1}|Z_{1j}=1\right)$ for j=1,…,K and $\overset{P}{⟶}$ denotes the convergence in probability.*

The proof of Proposition 1 necessitates the following technical Lemma.

**Lemma** **1.** *Let ${\left(S_{n}\right)}_{n\geq 1}$ be a sequence of i.i.d. random variables in the space of square integral functions $L^{2}$ with a common mean μ and let ${\left(T_{n}\right)}_{n\geq 1}$ be a sequence of random variables. Hence,*

$$E\left({\bar{S}}_{n}|T_{n}\right)\underset{n\to +\infty }{\overset{L^{2}}{⟶}}\mu ,\;where\;{\bar{S}}_{n}=\frac{1}{n}\sum_{i=1}^{n}S_{i},$$

*where $L^{2}$ denotes the mean quadratic convergence $L^{2}$.*

The proof of this lemma is reported in [35].

**Proof of Proposition** **1.** First, using Lemma 1 and following the same steps as the proof of Theorem 4.4 in [35], we prove that ${\hat{\pi }}_{j}\underset{n\to +\infty }{\overset{P}{⟶}}\pi_{j},$ $\lim_{n\to \infty }{\hat{\mu }}_{j}\underset{n\to +\infty }{\overset{P}{⟶}}\mu_{j}$ and ${\hat{\sigma^{2}}}_{j}\underset{n\to +\infty }{\overset{P}{⟶}}\sigma_{j}^{2}.$ Then, according to Slutsky’s Theorem, we obtain

(18) $$\begin{array}{c} \sum_{k=1}^{K}{\hat{\pi }}_{k}N\left({\hat{\mu }}_{k},{\hat{\sigma }}_{k}\right)\left(x\right)\underset{n\to +\infty }{\overset{P}{⟶}}\sum_{j=1}^{K}\pi_{j}N\left(\mu_{j},\sigma_{j}^{2}\right)\left(x\right). \end{array}$$

Second, based on Theorem 3.1 in [16], we obtain

(19) $$\begin{array}{c} {\tilde{f}}_{1,n,m}\left(x\right)\underset{n\to +\infty }{\overset{P}{⟶}}f\left(x\right)\;\mathrm{for}\;x\in \left[0,1\right]. \end{array}$$

In addition, referring to (18) and (19) and grounded on the application of Slutsky’s Theorem, we conclude the proof.   □

According to [21], the density f(x) is a close approximation to the mixture density $f_{2}\left(x\right)$. Thus, the estimator ${\hat{f}}_{n,m}\left(x\right)$ provides an approximation to the true density f(x).

To study the asymptotic normality of the estimator ${\hat{f}}_{n,m}$ given by (17), we set forward the following assumptions in [36].

**(A1)**  For almost x∈[0,1] and for all i,j,h=1…,ν, the partial derivatives $\partial g/\partial \xi_{i}$, $\partial^{2}g/\partial \xi_{i}\partial \xi_{j}$ and $\partial^{3}g/\partial \xi_{i}\partial \xi_{j}\partial \xi_{h}$ of the density *g* exist and satisfy that $\left|\frac{\partial g(x|\theta )}{\partial \xi_{i}}\right|,$$\left|\frac{\partial^{2}g\left(x|\theta \right)}{\partial \xi_{i}\xi_{j}}\right|$ and $\left|\frac{\partial^{3}g\left(x|\theta \right)}{\partial \xi_{i}\xi_{j}\xi_{h}}\right|$ are bounded, respectively, by $J_{i}$, $J_{ij}$ and $J_{ijh}$, where $J_{i}$ and $J_{ij}$ are integrable and $J_{ijh}$, satisfies
  $$\int_{0}^{1}J_{ijh}\left(x\right)g\left(x|\hat{\theta }\right)dx<\infty .$$
**(A2)**  The Fisher information matrix I(θ) is positively defined at $\hat{\theta }$.

**Proposition** **2** (Normality asymptotic)**.** *Under the regularity conditions**(A1)**–**(A2)**, if f(x)>0 for all x∈[0,1], 2≤m≤(n/logn) and $\lim_{n,m\to \infty }n^{2/3}/m=0$, then, we obtain*

$$n^{1/2}m^{-1/4}\left[{\hat{f}}_{n,m}\left(x\right)-\lambda f\left(x\right)-\left(1-\lambda \right)f_{2}\left(x\right)\right]\underset{n,m\to +\infty }{\overset{D}{⟶}}N\left(0,\lambda^{2}\gamma \left(x\right)\right),$$

*where $\gamma \left(x\right)=f\left(x\right){\left(4\pi x\left(1-x\right)\right)}^{-1/2},$ for x∈]0,1[, and $\overset{D}{⟶}$ denotes the convergence in distribution.*

**Proof of Proposition** **2.** Using Theorem 3.2 in [16], we obtain

$$n^{1/2}m^{-1/4}\left({\tilde{f}}_{1,n,m}\left(x\right)-f\left(x\right)\right)\underset{n,m\to +\infty }{\overset{D}{⟶}}N\left(0,\gamma \left(x\right)\right).$$

Thus,

$$n^{1/2}m^{-1/4}\left(\lambda {\tilde{f}}_{1,n,m}\left(x\right)-\lambda f\left(x\right)\right)\underset{n,m\to +\infty }{\overset{D}{⟶}}N\left(0,\lambda^{2}\gamma \left(x\right)\right).$$

According to Theorem 3.1 in [36], we obtain $\sqrt{n}\left(\hat{\theta }-\theta \right)\underset{n\to +\infty }{\overset{D}{⟶}}N\left(0,I{\left(\theta \right)}^{-1}\right).$ Using the delta method, we obtain

$$\sqrt{n}\left({\tilde{f}}_{2,n}\left(x|\hat{\theta }\right)-f_{2}\left(x|\theta \right)\right)\underset{n\to +\infty }{\overset{D}{⟶}}N\left(0,Df_{2}\left(x|\theta \right)I{\left(\theta \right)}^{-1}Df_{2}{\left(x|\theta \right)}^{T}\right),$$

where $Df_{2}\left(x|\theta \right)$ is the Jacobian matrix of $f_{2}\left(x|\theta \right)=f_{2}\left(x\right)$ and ${\tilde{f}}_{2,n}\left(x|\hat{\theta }\right)={\tilde{f}}_{2,n}\left(x\right)$. Since $m^{-1/4}\to 0$ if m→∞, then, using Slutsky’s Theorem, we conclude the proof. □

The following corollary is a consequence of the previous proposition which gives an asymptotic confidence interval of the density *f*, for a risk α∈]0,1[.

**Corollary** **1.** *The 100(1−α)% asymptotic confidence interval of f(x) is given by*

$$\left({\hat{f}}_{n,m}\left(x\right)\pm \frac{z_{1-\frac{\alpha }{2}}\lambda \sqrt{\gamma (x)}}{\sqrt{n}m^{-1/4}}\right),$$

*where $z_{1-\frac{\alpha }{2}}$ is the normal $\left(1-\frac{\alpha }{2}\right)$ quantile.*

In the next section, we study the performance of the proposed estimator in estimating different distributions by comparing it to the performances of the Bernstein estimator and of the Gaussian kernel estimator.

## 5. Numerical Studies

### 5.1. Comparison Study

In this section, we investigate the performance of the proposed estimator given in (17), through estimating different densities by comparing it to the performance of the Bernstein estimator defined in (2), the standard Gaussian kernel estimator defined in (1) and the Guan’s estimator defined in (3). We apply the Bernstein estimator when the sample is concentrated on the interval [0,1]. For this purpose, we need to make some suitable transformations in the different cases that are listed as follows:

**1.**  Let us suppose that *X* is concentrated on a finite support [a,b]; then, we work with the sample values $Y_{1},\ldots ,Y_{n}$, where $Y_{i}=\left(X_{i}-a\right)/\left(b-a\right)$.
**2.**  For the density functions concentrated on R, we can use the transformed sample $Y_{i}=1/2+\pi^{-1}\arctan\left(X_{i}\right)$, which transforms the range to the interval (0,1).
**3.**  For the support $R_{+}$, we can use the transformed sample $Y_{i}=X_{i}/\left(1+X_{i}\right)$, which transforms the range to the interval (0,1).

If the support is infinite, say, R=(−∞,∞), we can consider $\left[x_{1},x_{t}\right]\subset \left[a,b\right]$ as the finite support of *f*, where $x_{1}$ and $x_{t}$ are the minimum and the maximum order, respectively. We choose *a* and *b* such that F(a) and 1−F(b) are of $O(n^{-1})$, $a<x_{1}$, and $b>x_{t}$, where *F* is the distribution function [27]. Then, we can use the transformed sample, which transforms $\left[x_{1},x_{t}\right]$ to the interval [0,1] mentioned in the case 1.

In the simulation study, three sample sizes were considered, n=50, n=100, and n=200, as well as the following density functions:

**(a)**  The beta mixture density 0.5B(3,9)+0.5B(9,3);
**(b)**  The beta mixture density 0.5B(3,1)+0.5B(10,10);
**(c)**  The normal mixture density 1/4N(2,1)+3/4N(−3,1);
**(d)**  The chi-squared $\chi_{n}\left(2\right)$ density.
**(e)**  The gamma mixture density 0.5G(1,6)+0.5G(6,1);
**(f)**  The gamma mixture density 0.5G(1,2)+0.5G(4,2).

Our sample was decomposed into a learning sample of a size of 2/3 of the considered sample, on which the various statistical methods were constructed, and a second sample of a size of 1/3 of the considered sample, on which the predictive performance of the three methods were tested. For each density function *f* and sample size *n*, we computed the integrated squared error (ISE), the integrated absolute error (IAE) and the Kullback–Leibler divergence (KL) of the estimator ${\hat{f}}_{n,m}$ over N=500 trials.

$$\hat{ISE}=\frac{1}{N}\sum_{k=1}^{N}ISE\left({\hat{f}}_{k}\right),\;\hat{IAE}=\frac{1}{N}\sum_{k=1}^{N}IAE\left({\hat{f}}_{k}\right)\;\mathrm{and}\;\hat{KL}=\frac{1}{N}\sum_{k=1}^{N}KL\left({\hat{f}}_{k}\right),$$

where ${\hat{f}}_{k}$ is the estimator computed from the kth sample and

$$\begin{array}{ccc} ISE[{\hat{f}}_{k}] & = & \int_{0}^{1}{\left({\hat{f}}_{k}\left(x\right)-f\left(x\right)\right)}^{2}dx,\;IAE\left({\hat{f}}_{k}\right)=\int_{0}^{1}\left|{\hat{f}}_{k}\left(x\right)-f\left(x\right)\right|dx,\\ KL({\hat{f}}_{k}|f) & = & \int_{0}^{1}{\hat{f}}_{k}\left(x\right)\log\frac{{\hat{f}}_{k}\left(x\right)}{f(x)}dx. \end{array}$$

Indeed, it is advised to consider a learning sample bigger than a testing sample. In this work, our sample was decomposed into a learning sample of a size of 2/3 of the considered sample, on which the various statistical methods were constructed, and a second sample of a size of 1/3 of the considered sample, on which the predictive performances of the three methods were tested. Each run of the proposed estimator performed the following steps:

**-**  We first generated a random sample ${\left(X_{i}\right)}_{1\leq i\leq n}$ of size *n* from the models’ density (a)−(f).
**-**  We then split the generated data into a training set of a size of 2/3 of the considered sample and a test set of a size of 1/3 of the considered sample.
**-**  We applied the proposed estimator, using the observed data $X_{i}$ only from the training set, in order to estimate the density function.
**-**  The test set was then used to compute the estimation errors $\hat{ISE}$, $\hat{IAE}$ and $\hat{KL}$.

To select the optimal parameter *K*, we used the Gap Statistics algorithm [37]. We considered a Monte Carlo experiment to select the optimal choice of the degree *m* of the Bernstein polynomial and the bandwidth *h* of the kernel estimator, for each point x∈[0,1]. We determined the parameters *m* (for 1≤m≤300) and *h* (for h=i/1000 with 1≤i≤300), which minimized the ISE, which was approximated by the $\hat{ISE}$.

We considered N=500 random samples of sizes n=50, n=100 and n=200.

**Figure 1:**
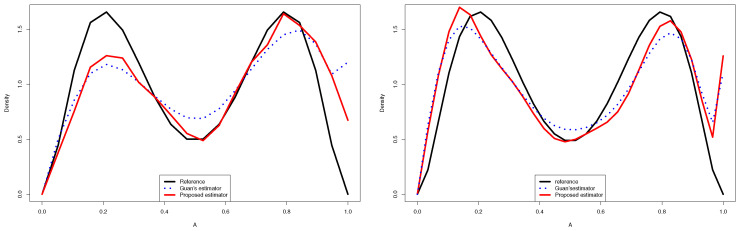
Quantitative comparison between the proposed estimator and Guan’s estimator of 0.5B(3,9)+0.5B(9,3) for n=50 (**left**) and n=100 (**right**).

**Figure 2:**
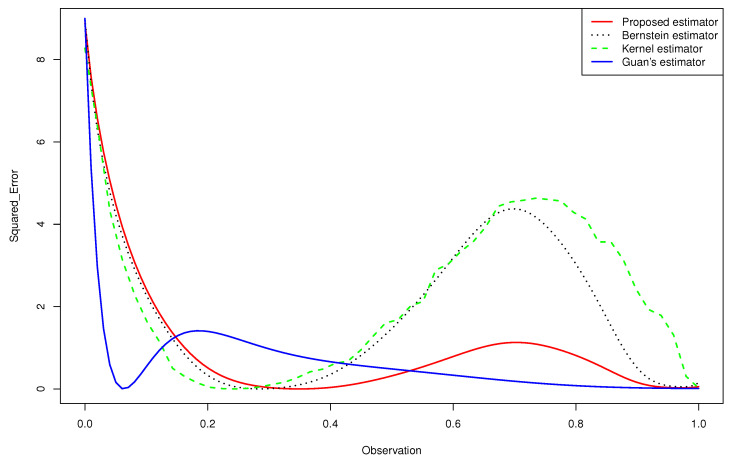
Quantitative comparison among the mean squared error of the kernel estimator, the Bernstein estimator, the Guan’s estimator and the proposed estimator of 0.5G(1,6)+0.5G(6,1) for n=200.

**Figure 3:**
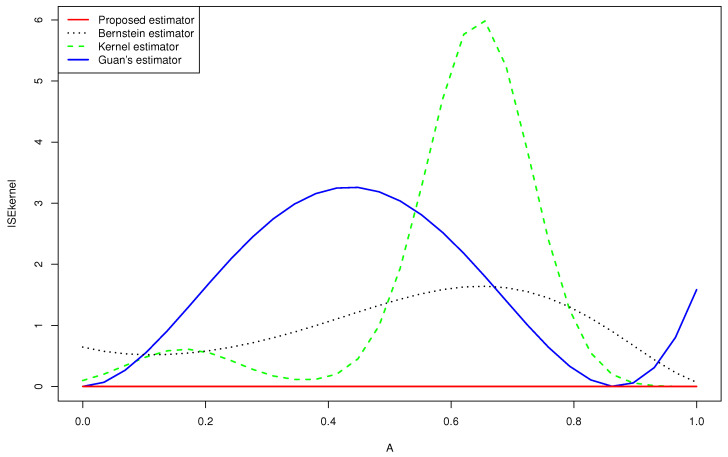
Quantitative comparison among the mean squared error of the kernel estimator, the Bernstein estimator, the Guan’s estimator and the proposed estimator of 0.25N(2,1)+0.75N(−3,1) for n=200.

Departing from Table 1, Table 2 and Table 3 and Figure 1, we deduce the following:

**-**  The results displayed in Table 1, Table 2 and Table 3 show that the $\hat{ISE}$, $\hat{IAE}$ and $\hat{KL}$ decreased as the sample size increased.
**-**  Using the proposed estimator, we obtained better results than those given by the other estimators in a large part of the cases.
**-**  The Figure 2 and 3 give a better sense of where the error is located.
**-**  For the case (e) of the gamma mixture, the average $\hat{ISE}$ and $\hat{IAE}$ of Guan’s estimator (1.3) were smaller than those obtained by the proposed density estimator (3.4) and the Bernstein estimator (1.2). However, in all the other cases, using an appropriate choice of the degree *m*, the average $\hat{ISE}$ and $\hat{IAE}$ of the proposed density estimator (3.4) were smaller than what achieved by the kernel estimator (1.1), the Bernstein estimator (1.2) and Guan’s estimator (1.3), even when the sample size was large for same cases.
**-**  When we changed the parameters of the gamma mixture density in the sense that we had a smaller bias, our estimator was more competitive than the other approaches and we obtained better results.
**-**  Almost in all considered cases, the average $\hat{KL}$ of the density estimator (17) was smaller than that obtained by the Bernstein estimator defined in (2), that of the kernel estimator defined in (1) and that of Guan’s estimator.
**-**  In the considered distribution 0.5B(3,9)+0.5B(9,3), by choosing the appropriate *m*, the curve of the proposed distribution estimator (3.4) was closer to the true distribution than that of Guan’s estimator (1.3), even when the sample size was very large.

Referring to Figure 2 and Figure 3, we infer the following:

**-**  None of the estimators for the gamma mixture density 0.5G(6,1)+0.5G(1,6) had good approximations near x=0. However, the $\hat{ISE}$ of the proposed estimator was closer to zero than that of the Bernstein estimator and the kernel estimator, especially near the edge x=1.
**-**  Guan’s estimator and the kernel estimator for the normal mixture density 0.25N(2,1)+0.75N(−3,1) had good approximations near x=0. However, the $\hat{ISE}$ of the proposed estimator was closer to zero than that of the other estimators, especially near the two edges.

Therefore, we note that, for difficult distributions that diverge at the boundaries, the proposed method would fail, but not as badly as the standard methods without shrinkage. In addition, the performed simulations revealed that, on average, the proposed approach could lead to satisfactory estimates near the boundaries, better than the classical Bernstein estimator.

### 5.2. Real Dataset

#### 5.2.1. COVID-19 Data

In this subsection, we consider the COVID-19 data displayed in the INED website https://dc-covid.site.ined.fr/fr/donnees/france/ (accessed on 16 February 2022). These data concern the numbers of deaths due to COVID-19 in France (daily) from 21 March 2021, for 454 days. These data are such that $\min_{i}\left(x_{i}\right)=605$ and $\max_{i}\left(x_{i}\right)=0$. Then, it is convenient to assume that the density of the numbers of deaths is defined on the interval [0,605] and transform the data into the interval unit. The Monte Carlo procedure was performed and resulted in h=0.07659612 for the standard kernel estimator defined in (1.1), $m_{1}=20$ for the Bernstein estimator defined in (1.2), the proposed estimator, and $m_{2}=12$ for Guan’s estimator. These estimators are exhibited in Figure 1 (right panel) along with a histogram of the data. All the estimators are smooth and seem to capture the pattern highlighted by the histogram. We record that the proposed estimator outperformed the other estimators near the boundaries.

#### 5.2.2. Tuna Data

**Figure 4:**
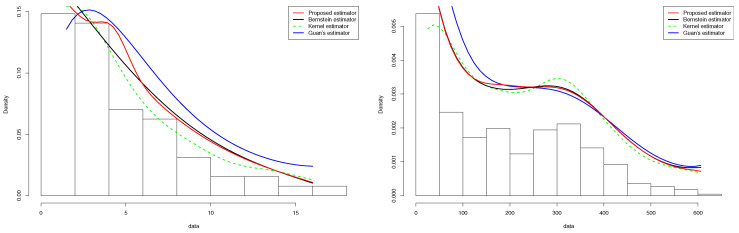
Qualitative comparison among the kernel estimator defined in (1), the Bernstein estimator defined in (2), Guan’s estimator (3) and the proposed density estimator (17) of Tuna data (**left**) and of COVID-19 data (**right**).

The last example concerns the tuna data reported in [38]. The data are derived from an aerial line transect survey of Southern Bluefin Tuna in the Great Australian Bight. An aircraft with two spotters on board flew randomly over allocated line transects. These data correspond to the perpendicular sighting distances (in miles) of 64 detected tuna schools to the transect lines. The survey was conducted in summer when tuna data tend to stay on the surface. The data are such that $\min_{i}\left(x_{i}\right)=0.19$ and $\max_{i}\left(x_{i}\right)=16.26$. The Monte Carlo procedure was performed and resulted in h=0.1079 for the standard kernel estimator defined in (1), $m_{1}=13$ for the Bernstein estimator defined in (2) and the proposed estimator, and $m_{2}=6$ for Guan’s estimator. These estimators are illustrated in Figure 4 (left panel) along with a histogram of the data. All the estimators are smooth and seem to capture the pattern highlighted by the histogram. We assert that the proposed estimator outperformed the other estimator, especially near the boundaries.

## 6. Conclusions

In this paper, we propose a shrinkage estimator of a density function based on the Bernstein density estimator and using a finite Gaussian mixture density. This method rests on three steps. The first step consists of considering the Bernstein estimator ${\tilde{f}}_{1,n,m}$. The second relies upon the Gaussian Mixture density ${\tilde{f}}_{2,n}$ as an estimator of the unknown density *f*. The last step consists of considering the shrinkage form $\lambda {\tilde{f}}_{1,n,m}+\left(1-\lambda \right){\tilde{f}}_{2,n}$ and EM algorithm in order to estimate the parameter λ. The asymptotic properties of this estimator were established. Afterwards, we demonstrate the effectiveness of the proposed method using some simulated and real data. We clarify how it can lead to very satisfactory estimates near the boundaries and in terms of ISE, IAE and KL. Eventually, we would simply assert that our research work is a step that may be taken further, extended and built upon as it lays the ground and paves the way for future works to elaborate a semi-parametric regression estimator using the shrinkage method. We also plan to work on the case where λ is a random variable. Another future research direction would be to extend our findings to the setting of serially dependent observations.

## Figures and Tables

**Table 1:** Average $\hat{ISE}$ for N=500 trials of Bernstein estimator, standard Gaussian kernel estimator and the proposed estimator ${\hat{f}}_{n,m}$, for n=50, n=100 and n=200. The bold values indicate the smallest values of ISE.

Density	*n*	Proposed	Bernstein	Kernel	Guan’s
		Estimator	Estimator	Estimator	Estimator
	50	0.092242	0.096684	0.197497	0.140323
(a)	100	0.091364	0.092242	0.174251	0.089129
	200	0.075532	0.079299	0.148143	0.086305
	50	1.157827	1.215347	0.530446	0.816906
(b)	100	0.235402	0.306704	0.482152	0.276573
	200	0.199786	0.289870	0.474805	0.255716
	50	0.001423	1.808252	2.222369	1.035522
(c)	100	0.000384	1.410606	1.641689	1.014602
	200	0.000227	1.348292	1.077352	0.994346
	50	0.525192	2.812448	4.936701	0.589465
(d)	100	0.492752	2.483141	2.331765	0.579595
	200	0.162917	0.898103	1.154646	0.507362
	50	2.180849	2.231986	2.424656	1.084340
(e)	100	2.050098	2.133496	2.295932	0.835670
	200	2.042379	2.086204	2.053453	0.717715
	50	0.313388	0.896995	1.397111	0.663889
(f)	100	0.253988	0.656400	0.762742	0.516530
	200	0.186290	0.577408	0.417980	0.472094

**Table 2:** Average $\hat{IAE}$ for N=500 trials of Bernstein estimator, standard Gaussian kernel estimator and the proposed estimator ${\hat{f}}_{n,m}$, for n=50, n=100 and n=200. The bold values indicate the smallest values of IAE.

Density	*n*	Proposed	Bernstein	Kernel	Guan’s
		Estimator	Estimator	Estimator	Estimator
	50	0.250241	0.250241	0.391072	0.251641
(a)	100	0.196423	0.207109	0.367361	0.232399
	200	0.180536	0.191673	0.348499	0.214117
	50	0.855008	0.823416	0.621137	0.747562
(b)	100	0.417735	0.457722	0.669027	0.438088
	200	0.386280	0.455983	0.583057	0.423595
	50	0.035161	0.971720	1.238839	0.948451
(c)	100	0.019331	0.960044	1.157838	0.944000
	200	0.013233	0.923259	0.953675	0.931293
	50	0.669269	0.807667	1.942776	0.648617
(d)	100	0.657815	1.486974	1.415036	0.642986
	200	0.351205	1.573886	0.881866	0.637942
	50	0.830317	0.834203	1.499828	0.678362
(e)	100	0.706770	0.713225	1.374609	0.645112
	200	0.681767	0.692357	1.225502	0.620315
	50	0.453948	0.640596	1.068483	0.608421
(f)	100	0.414676	0.714844	0.782939	0.584572
	200	0.383248	0.883320	0.516527	0.584320

**Table 3:** Average $\hat{KL}$ for N=500 trials of Bernstein estimator, standard Gaussian kernel estimator and the proposed estimator ${\hat{f}}_{n,m}$, for n=50, n=100 and n=200. The bold values indicate the smallest values of KL.

Density	*n*	Proposed	Bernstein	Kernel	Guan’s
		Estimator	Estimator	Estimator	Estimator
	50	0.025048	0.025048	0.289818	0.066817
(a)	100	0.012086	0.015023	0.081830	0.058541
	200	0.003256	0.001788	0.060468	0.029419
	50	1.088053	1.120246	0.575298	0.667079
(b)	100	0.284795	0.325933	0.381406	0.659105
	200	0.255697	0.310759	0.150096	0.654338
	50	2.689781	3.871172	4.732324	3.360316
(c)	100	0.011844	3.591156	4.196295	3.356093
	200	0.009426	3.505050	3.169852	3.318666
	50	0.976450	0.870222	4.702359	0.878995
(d)	100	0.960633	1.572251	3.031783	0.763044
	200	0.281862	1.584022	1.537355	0.742696
	50	1.549035	1.560172	5.538142	1.799133
(e)	100	1.207084	1.217043	3.498273	1.557894
	200	1.153420	1.169645	1.322299	1.387843
	50	0.528337	1.052789	1.952294	0.651422
(f)	100	0.292017	0.805962	1.191070	0.537625
	200	0.062893	0.589790	0.679154	0.339442

## Data Availability

Not applicable.
